# IRON magnetic resonance: a robust technique for angiography providing high blood-to-tissue contrast within the clinically approved dosage of superparamagnetic nanoparticles

**DOI:** 10.1186/1532-429X-13-S1-P377

**Published:** 2011-02-02

**Authors:** Gitsios Gitsioudis, Matthias Stuber, Ingolf Arend, Moritz Thomas, Jing Yu, Thomas Hilbel, Evangelos Giannitsis, Hugo A Katus, Grigorios Korosoglou

**Affiliations:** 1 University Clinic of Heidelberg, Heidelberg, Germany; 2 Russell H. Morgan Department of Radiology and Radiological Sciences, Division of MR Research, Johns Hopkins University School of Medicine, Baltimore, MD, USA; 3 University of Applied Sciences Gelsenkirchen, Department of Physical Engineering, Gelsenkirchen, Germany; 4 Russell H. Morgan Department of Radiology and Radiological Sciences, Division of MR Research, Johns Hopkins University School of Medicine, , Maryland, USA, Baltimore, MD, USA

## Introduction

Conventional MR angiography (MRA) exploits the property of exogenous contrast agents to shorten the T1 relaxation time. However, undesirable signal from surrounding tissue due to T1 recovery remains. IRON MRA is a promising off-resonance imaging technique, and provides in conjunction with superparamagnetic nanoparticles high intravascular contrast between the blood-pool and background tissue without need for image subtraction while T1 recovery of the surrounding tissue is no longer a problem.

## Purpose

Experiments were approved by the institutional animal care committee. Twelve rabbits were imaged at baseline and serially after the administration of 10 incremental dosages of 0.57 mg Fe (P904)/Kg. Conventional T1-weighted and IRON MRA were performed on a clinical 1.5-T imager. We performed angiography of the thoracic aorta (n=6), and angiography of the abdominal aorta and peripheral vessels (n=6). Contrast-to-noise ratios (CNR) were obtained in the thoracic and abdominal aorta of the rabbits and vessel sharpness was quantified in peripheral iliac and femoral vessels.

## Results

**Figure 1:**
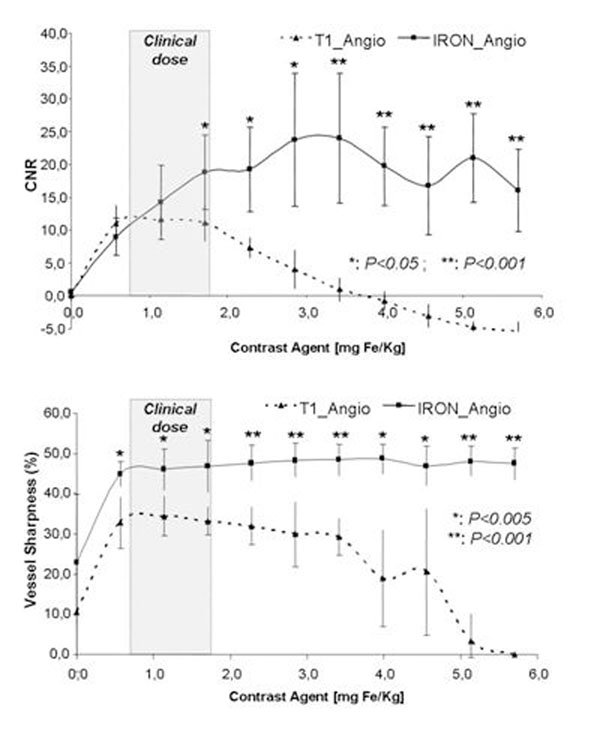


**Figure 2:**
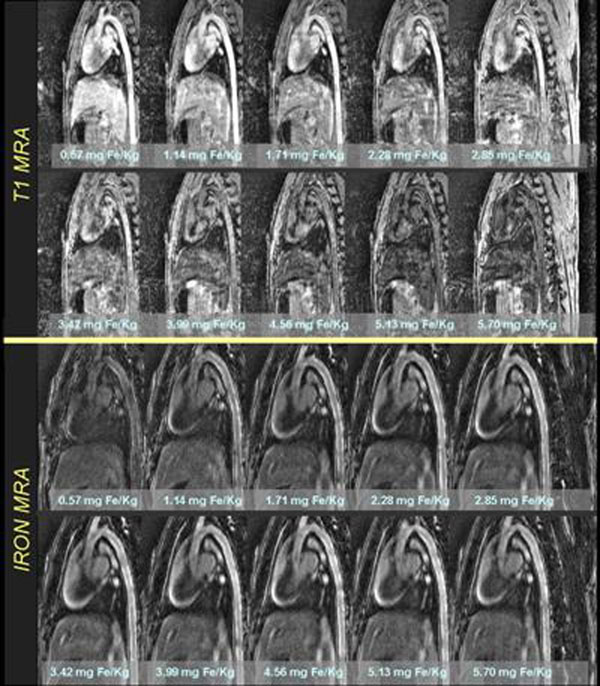


With T1 weighted angiography, CNR in the thoracic and abdominal aorta and vessel sharpness in peripheral vessels initially increased up to a dosage of (~1 mg Fe/Kg), whereas subsequently, a decrease in signal was observed as a function of further dose increase. In contrast, using IRON MRA, CNR and vessel sharpness both progressively increased during incremental administration of the contrast agent (Figure 1). CNR and vessel sharpness were both significantly higher within the range of the clinically approved dosage of the contrast agent (CNR of 11.1 versus 18.8 and vessel sharpness of 33.2% versus 46.8% for the dose of 1.71 mg Fe/Kg, respectively, both p<0.05 for both). For IRON MRA both CNR and vessel sharpness continued to increase as a function of the contrast agent concentration. A representative example of the thoracic aorta imaged using T1 weighted and IRON MRA can be appreciated in Figure 2.

## Conclusions

IRON MRA provides significantly higher blood-to-tissue contrast both in the aorta and in small peripheral rabbit vessels compared to T1-weigthed angiography within the dose range of approved for clinical human use. High CNR and vessel sharpness remain stable over a very wide range of contrast agent concentrations, which may possibly allow for more robust MRA acquisitions in clinical applications.

